# V Aortic Arch Remnant

**DOI:** 10.3390/diagnostics15081036

**Published:** 2025-04-18

**Authors:** Corrado Tagliati, Marco Fogante, Stefania Lamja, Cecilia Cerimele, Alessia Quaranta, Alfonso Alberto Matarrese, Davide Battista, Antonio Bernardini, Giulio Argalia, Iacopo Carbone, Ernesto Di Cesare, Nicolò Schicchi, Giuseppe Lanni

**Affiliations:** 1 AST Ancona, Ospedale di Comunità Maria Montessori di Chiaravalle, Via Fratelli Rosselli 176, 60033 Chiaravalle, Italy; corrado.tagliati@gmail.com; 2 Maternal-Child, Senological, Cardiological Radiology and Outpatient Ultrasound, Department of Radiological Sciences, University Hospital of Marche, Via Conca 71, 60126 Ancona, Italy; giulio.argalia@gmail.com (G.A.); nicolo.schicchi@ospedaliriuniti.marche.it (N.S.); 3 Department of Biotechnological and Applied Clinical Sciences, University of L’Aquila, Via Vetoio, 67100 L’Aquila, Italy; stefanialamja@gmail.com (S.L.); ernesto.dicesare@univaq.it (E.D.C.); 4 Department of Services, UOSD Radiology, San Liberatore Hospital, Viale Risorgimento, 64032 Atri, Italy; cec.cerimele@gmail.com (C.C.); davide.battista@aslteramo.it (D.B.); 5 AST Macerata, Cardiologia, Distretto Sanitario di Civitanova Marche, Via Abruzzo, 62012 Civitanova Marche, Italy; alessiaquaranta84@gmail.com; 6 AST Ascoli Piceno, Cardiologia, Ospedale Mazzoni, Via degli Iris 1, 63100 Ascoli Piceno, Italy; alfonsomatarrese@gmail.com; 7 Department of Services, UOSD Diagnostica per Immagini Teramo, Ospedale Civile Giuseppe Mazzini, Piazza Italia, 64100 Teramo, Italy; antonio.bernardini@aslteramo.it (A.B.); giuseppe.lanni@aslteramo.it (G.L.); 8 Department of Radiological, Oncological and Pathological Sciences, Academic Diagnostic Imaging Division, I.C.O.T. Hospital, Sapienza University of Rome, Via F. Faggiana 1668, 04100 Latina, Italy; iacopo.carbone@uniroma1.it

**Keywords:** computed tomography, aorta, thoracic, fifth aortic arch, remnant, congenital anomaly, type A, Weinberg classification, type A1, improved classification

## Abstract

Here, we describe the case of an asymptomatic 70-year-old male patient who performed a contrast-enhanced computed tomography examination for prostate cancer staging, and an exceptional finding was reported. Specifically, a probable and never before reported minimal V aortic arch remnant with a thin intima–media band that joins together the anterior and posterior aortic walls.

**Figure 1:**
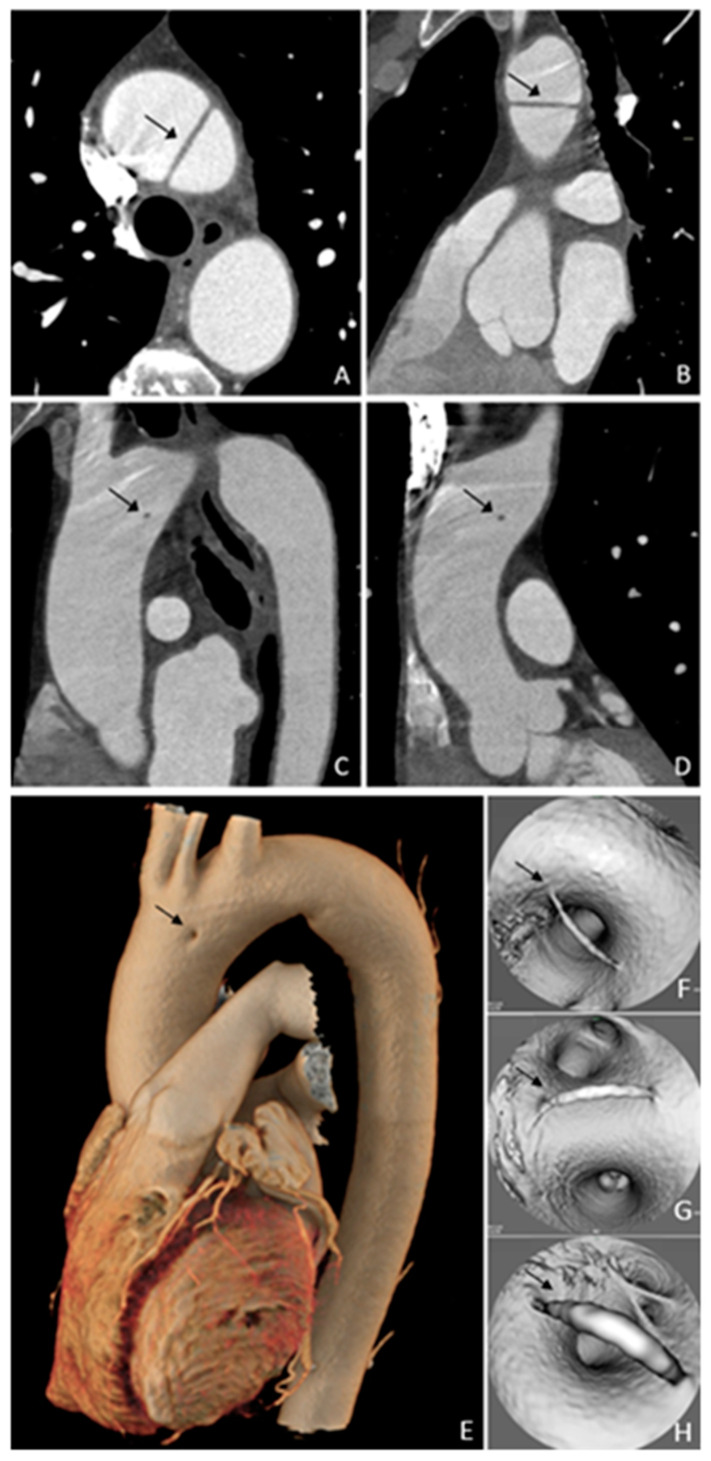
Axial (**A**), oblique sagittal (**B**,**C**), and oblique coronal (**D**) computed tomography images in an asymptomatic 70-year-old male patient who performed a computed tomography examination for prostate cancer staging. Images (**A**,**B**) could be misinterpreted as type A aortic dissection. However, looking at the oblique images (**C**,**D**), is evident that this is not the case—in fact, these images show that a thin line connects the anterior and posterior aortic walls, and it seems to be constituted by intima and media layers. Volume rendering (**E**) shows a small area with absent contrast enhancement, and the virtual endoluminal images (**F**,**G**,**H**) clearly show the stripe connecting the opposing walls. This seems to be a congenital anomaly due to a minimally imperfect aortic arch formation, and it could be a fifth aortic arch remnant. The patient underwent a cardiac surgery visit, and no follow-up was scheduled. The patient did not show any symptoms related to this condition two years post the computed tomography examination. Previously published articles reported aortic arch anomalies, in particular persistent fifth aortic arch (PFAA), which [1,2,3,4] has been classified into three types by Weinberg. Subsequently, an improved classification divided PFAA into four types based on its anatomical origin and hemodynamic changes. In particular, the imaging findings in our case suggest that it could belong to a previously unreported subtype of type A of the Weinberg classification and type A1 of the improved classification, which are characterized by a double-lumen aortic arch where two parallel arches coexist, with the innominate, left common carotid, and left subclavian arteries arising from the more superior arch, probably as derivatives of the normal embryonic fourth branchial arch [3,5,6]. The persistence of the fifth aortic arch is usually of no hemodynamic significance and it is incidentally diagnosed when it is characterized by a double-lumen aortic arch without other vascular malformations [5]. To the best of our knowledge, no previously published article has reported such an image of the thoracic aorta, so this case can expand the spectrum of aortic arch anomalies.

## Data Availability

Data are contained within the article.
